# Characterization of Resting-State Striatal Differences in First-Episode Depression and Recurrent Depression

**DOI:** 10.3390/brainsci12121603

**Published:** 2022-11-23

**Authors:** Jifei Sun, Zhongming Du, Yue Ma, Chunlei Guo, Shanshan Gao, Yi Luo, Qingyan Chen, Yang Hong, Xue Xiao, Xue Yu, Jiliang Fang

**Affiliations:** 1 Guang’anmen Hospital, China Academy of Chinese Medical Sciences, Beijing 100053, China; 2 Dongzhimen Hospital, Beijing University of Chinese Medicine, Beijing 100700, China; 3 Beijing First Hospital of Integrated Chinese and Western Medicine, Beijing 100026, China

**Keywords:** recurrent depressive episode, first depressive episode, functional connectivity, striatal, rs-fMRI

## Abstract

The presence of reward deficits in major depressive disorder is associated with abnormal striatal function. However, differences in striatal whole-brain functional between recurrent depressive episode (RDE) and first-episode depression (FDE) have not been elucidated. Thirty-three patients with RDE, 27 with FDE, and 35 healthy controls (HCs) were recruited for this study. A seed-based functional connectivity (FC) method was used to analyze abnormalities in six predefined striatal subregion circuits among the three groups of subjects and to further explore the correlation between abnormal FC and clinical symptoms. The results revealed that compared with the FDE group, the RDE group showed higher FC of the striatal subregion with the left middle occipital gyrus, left orbital area of the middle frontal gyrus, and bilateral posterior cerebellar gyrus, while showing lower FC of the striatal subregion with the right thalamus, left inferior parietal lobule, left middle cingulate gyrus, right angular gyrus, right cerebellum anterior lobe, and right caudate nucleus. In the RDE group, the HAMD-17 scores were positively correlated with the FC between the left dorsal rostral putamen and the left cerebellum posterior lobe. This study provides new insights into understanding the specificity of striatal circuits in the RDE group.

## 1. Introduction

Major Depressive Disorder (MDD), as a common clinical psychiatric disorder, causes great distress to patients [1]. Epidemiological surveys have shown that there are approximately 350 million people with MDD worldwide, and the condition is expected to become the number one burden disease worldwide by 2030 [2,3]. According to the ICD-10 classification criteria, MDD can be classified as first depressive episode (FDE) and recurrent depressive disorder (RDE) [4]. Despite numerous studies, patients with first-episode MDD still have a risk of recurrence of up to 75–90% after receiving treatment [5]. On average, patients with a history of MDD will have between five and nine episodes of varying degrees of MDD in their lifetime, placing a heavy burden on the individual and society [6]. Therefore, understanding the different pathogeneses of RDE and FDE is essential to understand the pathogenesis of MDD at different stages.

Both FDE and RDE are clinically common subtypes of MDD, but their clinical symptoms may differ [4]. Previous studies have found that RDE has a higher severity and incidence of depression and somatic symptoms, more severe cognitive impairment, and an increase in symptom severity with the number of episodes compared to FDE [7,8]. It has also been found that RDE is more severe co-morbid medical disease, with worse prognoses, more residual symptoms, and lower quality of life compared to the FDE [9,10]. Therefore, there may be different physio-pathogeneses for the different clinical symptoms of RDE and FDE.

Resting-state functional magnetic resonance imaging (rs-fMRI) is an effective tool for studying psychiatric disorders, including MDD [11,12], autism [13], schizophrenia [14], and bipolar disorder [15]. Rs-fMRI was also further applied in the study of subtype MDD [16,17]. Functional connectivity (FC) is a common method used in rs-fMRI to study the temporal correlation between brain regions, reflecting the functional integration of brain regions during brain activity [18,19]. Previous studies have shown that the presence of FC abnormalities in MDD patients [20,21,22]. In addition, it has also been shown that RDE has anomalies in the default mode network (DMN), cognitive control network (CCN) and salience network [23,24,25].

In addition, we found that neurological dysfunction in MDD patients also focused on abnormalities in the cortico-striato-thalamo-cortical (CSTC) circuit [26,27]. The striatum, an important part of the CSTC, is highly functionally heterogeneous and is associated with psychomotor retardation and pleasure deficits, it is also an important component of the reward network [28,29,30,31]. Pleasure deficit is a core symptom of MDD and reflects a deficit in the functioning of the reward system [29,32]. The striatum is involved in the secretion of dopamine in the body and its dysfunction tends to affect the secretion of dopamine, which leads to reward deficits [33,34]. The internal structure of the striatum is specific and includes the caudate, putamen and the ventral striatum (VS) [35,36]. The dorsal caudate receives projections from the dorsal prefrontal cortex (PFC) and is associated with cognitive division functions, the putamen receives inputs from the anterior cingulate cortex (ACC) and primary sensorimotor cortices and is associated with cognitive and motor functions, while the VS receives projections from the medial PFC and limbic structures and is associated with emotional functions [37,38]. It has been found that different functional states of the striatum exist at different stages of MDD [38]. Therefore, it is necessary to study the striatal circuit of RDE and FDE, which is beneficial to further explore the differences in neuropathological mechanisms between them.

However, only one previous study on striatal aspects of MDD at different stages found that increased caudate-insular and reduced VS-cerebellar FC in remitted patients and first-degree relatives might be related to the disease itself and have potential for predicting risk for and recurrence of MDD [38]. Two other studies were conducted using task states to observe differences in striatal circuit between an rMDD group and a healthy control (HC) group [39,40]. One of these found that the FC of the striatal circuit during stress was higher in the rMDD group, suggesting that this may be a characteristic marker of MDD [39]. Another study found hypoactivation and impaired cognition in the putamen and caudate during a task state of working memory in the rMDD group, suggesting that MDD causes neuronal scar formation [40]. However, little is known about the differences between RDE and FDE in terms of striatal FCs.

Therefore, we used FC to observe the difference in brain functional activity between RDE and FDE in striatal subregions and further observed the correlation between FC abnormalities and clinical symptoms. This study will contribute to the understanding of neuroimaging markers for the onset and progression of MDD at different disease stages, and provide some insights into the differences between RDE and FDE in terms of reward network activity.

## 2. Methods

### 2.1. Participants

In this study, MDD patients were sourced from Guang’anmen Hospital, China Academy of Chinese Medical Science, Beijing First Hospital of Integrated Chinese and Western Medicine. Patients underwent a psychiatrist with extensive clinical experience for inclusion. Sixty patients with MDD were included according to the Diagnostic and Statistical Manual of Mental Disorders Fifth Edition criteria. The severity of the MDD patients recruited was generally moderate to severe. The inclusion criteria met the following [17]: (1) 17-item Hamilton Rating Scale for Depression (HAMD-17) score > 17; (2) age 18–60 years; (3) right-handedness; (4) The FDE group was a first episode with no previous antidepressant medication. The RDE group was previously in remission with antidepressants or other therapies, now relapsed, and did not receive any treatment in the 1 month prior to enrollment. Meanwhile, we recruited 35 age- and sex-matched HCs using an advertising format. The inclusion criteria met the following: (1) HAMD-17 score < 7; (2) age 18–60 years; (3) right-handedness.

The exclusion criteria for all subjects were as follows [17]: (1) contraindications to MRI scanning; (2) suicidal tendencies and thoughts, or other mental illness; (3) traumatic brain injury, tumor, or other cardiovascular or cerebrovascular disease; (4) lactating or pregnant status; (5) history of alcohol addiction.

### 2.2. Scan Acquisition

All subjects underwent MRI (Magneton Skyra 3.0 T, Siemens, Germany) scans at the Department of Radiology, Guang’anmen Hospital, China Academy of Chinese Medical Sciences. The subjects were instructed to keep their eyes closed and remain quiet and awake during the scan. A T2-weighted MRI plain scan was first performed to exclude organic brain lesions. Functional brain MRI data acquisition method: 3D T1-weighted imaging scan first, with the following parameters: time repetition (TR) = 2530 ms, time echo (TE) = 2.98 ms, field of view (FOV) = 256 mm × 256 mm, slice number = 48, slices = 192, flip angle (FA) = 7°, slice thickness = 1 mm, scanning time = 6 min 3 s. The blood oxygen level dependent (BOLD) sequence collects functional data with the following parameters: TR = 2000 ms, TE = 30 ms, FA = 90°, FOV= 240 mm × 240 mm, matrix = 64 × 64, number of obtained volumes = 200, slice thickness/spacing = 3.0/1 mm, scanning time = 6 min 40 s.

### 2.3. Image Processing

#### 2.3.1. fMRI Data Preprocessing

Data preprocessing was performed on rs-fMRI data based on MATLAB 2020a using the DPARSF 5.0 toolkit (DPARSF 5.0, http://www.rfmri.org/DPARSF, accessed on 15 September 2022) [41], with the following main steps: (1) DICOM raw data transfer to NIFTI format; (2) removal of the first 10 time points in order to retain stable signals; (3) slice timing; (4) head motion correction to the same position and provide data for later image quality control; (5) skull stripping, alignment, and segmentation by structural MRI; (6) evaluation of head rotation translation (quality control of no more than 2 mm translation and no more than 2° rotation in any direction); (7) all images were aligned to Montreal Neurological Institute (MNI) standard brain space using the EPI template, the images were smoothed with a smoothing parameter of full-width of half-maximum (FWHM) value of 6 mm; (8) linear detrending; (9) regression of head motion parameters, brain white matter signal and cerebrospinal fluid signal; (10) filtering (0.01–0.08 Hz).

#### 2.3.2. Seed-Based Functional Connectivity

**Figure 1:**
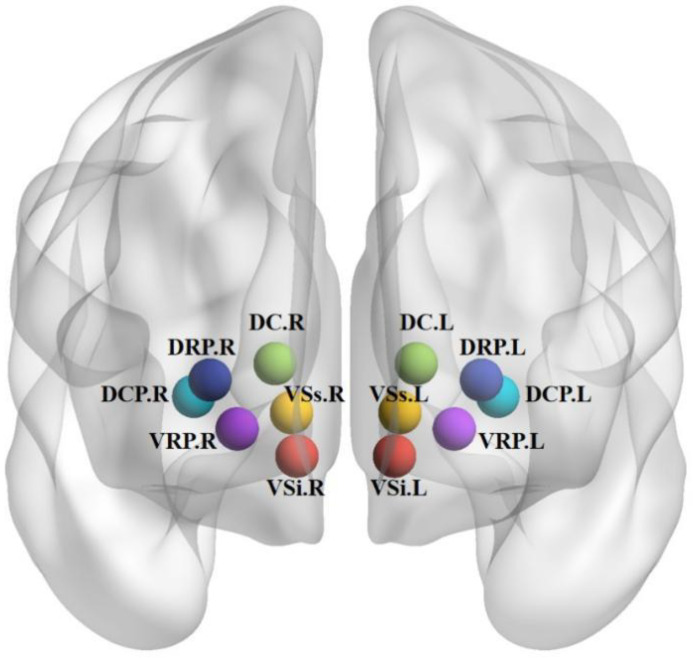
Definition of seeds in the striatal subregion. L, left; R, right; VSs, superior ventral striatum; VSi, inferior ventral striatum;DC, dorsal caudate; DRP, dorsal rostral putamen; DCP, dorsal caudal putamen; VRP, ventral rostral putamen.

The selection of seed point coordinates in the striatal subregion was based on previous studies [37,38,42]. Specifically, these were located (in MNI152 space) in the following areas: inferior VS (VSi) (x = ±9, y = 9, z = −8); superior VS (VSs) (x = ±10, y = 15, z = 0); dorsal caudate (DC) (x = ±13, y = 15, z = 9); dorsal caudal putamen (DCP) (x = ±28, y = 1, z = 3); dorsal rostral putamen (DRP) (x = ±25, y = 8, z = 6), and ventral rostral putamen (VRP) (x = ±20, y = 12, z = −3), with each seed covering 27 voxels in 2-mm^3^ space (radius = 4 mm). These six predefined striatal partitions are shown in detail in Figure 1.

The mean time series of all voxels in the ROI were extracted and calculated for each subject, and Pearson correlation analysis was done with the time series of whole-brain voxels to obtain a map of functional connectivity between the region of interest and the whole brain. The functional connectivity values were expressed as correlation coefficients, which were then transformed into Z values by Fisher’s Z-transformation to conform to a normal distribution.

### 2.4. Statistical Analyses

#### 2.4.1. Clinical Data Analysis

SPSS 23.0 software (IBM Corp, Released 2015, IBM SPSS Statistics for Windows, Version 23.0. Armonk, NY, USA) was used to statistically analyze the clinical data. The chi-square test was used to compare sex among the three groups. One-way analysis of variance (ANOVA) tests was used to compare age, yeas of education among the three groups. A two-sample *t*-test was used to compare HAMD-17 score, duration of illness between the two patient groups. The statistical threshold was set at *p* < 0.05 for statistical significance.

#### 2.4.2. fMRI Data Analysis

Image data statistics were analyzed using DPARSF 5.0 software (Released 2016,Beijing, China), and FC values between the three groups were analyzed using one-way ANOVA with sex, age, yeas of education and mean framewise displacement (FD) of the subjects among the three groups as covariates, and Gaussian random fields (GRF) correction was applied to the brain areas with differences in FC among the three groups, with cluster level *p* < 0.05, threshold voxel level *p* < 0.005 being defined as statistically significant differences. 

The mean FC values of the abnormal brain areas in the three groups were extracted using DPARSF software, and the results obtained were subjected to Bonferroni correction using SPSS 23.0 for post hoc two-group comparisons (RDE group vs. FDE group, RDE group vs. HC group, FDE group vs. HC group), and the differences were defined as statistically significant at *p* < 0.016 (0.05/3). Pearson correlation analysis was performed between the FC values of the three differential brain regions and HAMD-17 scores, and statistical significance was defined at *p* < 0.05.

## 3. Results

### 3.1. Characteristics of Research Samples

In this study, we did not find subjects with excessive FD. Thus, a total of 27 patients with FDE, 33 with RDE and 35 HCs met the criteria for this study. There were no statistical differences among the three groups in terms of age, sex, and years of education. In addition, we found no statistical difference between the RDE and FDE groups in terms of HAMD-17 scores, while there were statistical differences in the duration of illness (Table 1).

### 3.2. Among the Three Group Differences in Striatal FC and Post Hoc t-Test Analysis

#### 3.2.1. VSi, VSs

**Figure 2:**
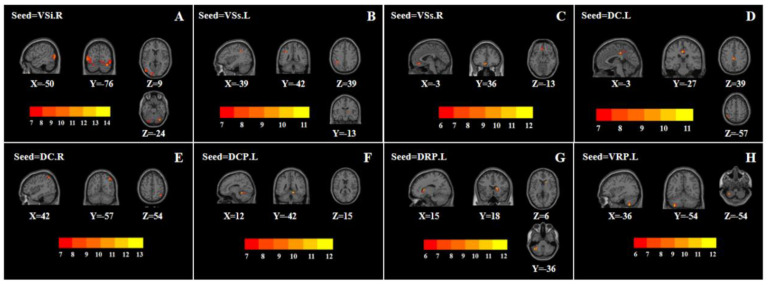
Brain regions with abnormal FC of the striatal subregion among the three groups based on one-way analysis of variance. The color bars indicate the *F*-value. L, left; R, right; VSs, superior ventral striatum; VSi, inferior ventral striatum; DC, dorsal caudate; DRP, dorsal rostral putamen; DCP, dorsal caudal putamen; VRP, ventral rostral putamen. (**A**), seed of the right VSi; (**B**), seed of the left VSs; (**C**), seed of the right VSs; (**D**), seed of the left DC; (**E**), seed of the right DC; (**F**), seed of the left DCP; (**G**), seed of the left DRP; (**H**), seed of the left VRP.

**Figure 3:**
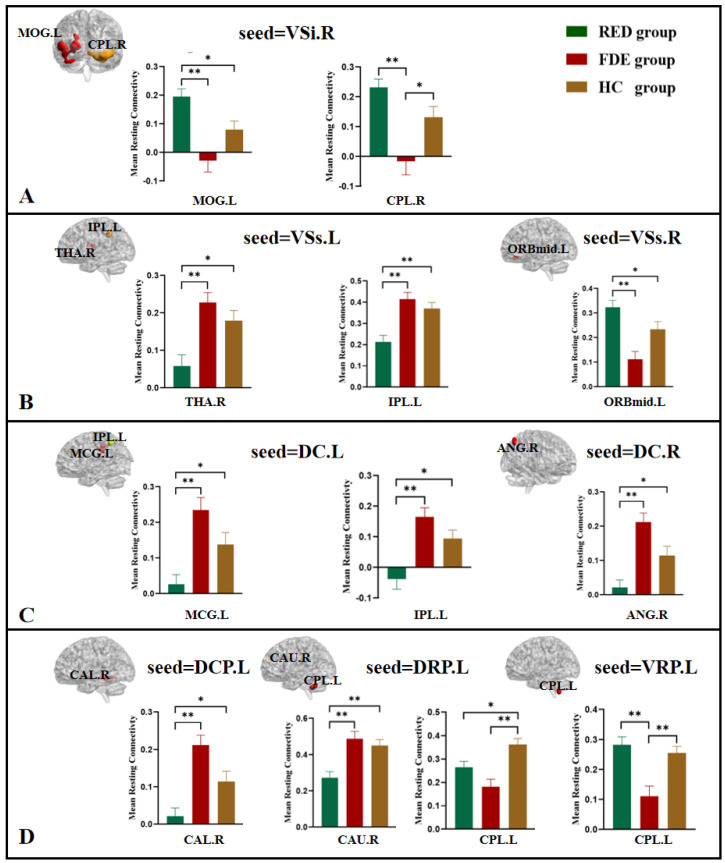
Post hoc two-sample *t*-tests (Bonferroni corrected) comparison showing FC values differences at peak voxel between each pair group (RDE group vs. FDE group, RDE group vs. HC group, FDE group vs. HC group). L, left; R, right; MOG, middle occipital gyrus; CPL, cerebellum posterior lobe; THA, thalamus; IPL, inferior parietal lobule; ORBmid, orbital area of the middle frontal gyrus; MCG, middle cingulate gyrus; ANG, angular; CAL, cerebellum anterior lobe; CAU, caudate. (**A**), seed of the right VSi; (**B**), seed of the left VSs and right VSs; (**C**), seed of the left DC and right DC; (**D**), seed of the left DCP, left DRP and left VRP. *, *p* < 0.016; **, *p* < 0.001.

One-way ANOVA showed that when the right VSi was used as the seed, the left middle occipital gyrus and right cerebellum posterior lobe were statistically significantly different among the three groups (Table 2, Figure 2A). Compared with the FDE group, the FC of the right VSi with the left middle occipital gyrus and right cerebellum posterior lobe was higher in the RDE group. Compared with the HC group, the FC of the right VSi with the left middle occipital gyrus was higher in the RDE group, and the FC of the right VSi with the right cerebellum posterior lobe was lower in the FDE group (Figure 3A).

One-way ANOVA showed that when the left VSs was used as the seed, the right thalamus and left inferior parietal lobule were statistically significantly different among the three groups (Table 2, Figure 2B). Compared with the FDE group, the FC of the left VSs with the right thalamus and left inferior parietal lobule was lower in the RDE group. Compared with the HC group, the FC of the left VSs with the right thalamus and left inferior parietal lobule was lower in the RDE group (Figure 3B).

One-way ANOVA showed that when the right VSs was used as the seed, the left orbital area of the middle frontal gyrus was statistically significantly different among the three groups (Table 2, Figure 2C). Compared with the FDE group, the FC of the right VSs with the left orbital area of the middle frontal gyrus was higher in the RDE group. Compared with the HC group, the FC of the right VSs with the left orbital area of the middle frontal gyrus was higher in the RDE group (Figure 3B).

#### 3.2.2. DC

One-way ANOVA showed that when the left DC was used as the seed, the left middle cingulate gyrus and left inferior parietal lobule were statistically significantly different among the three groups (Table 2, Figure 2D). Compared with the FDE group, the FC of the left DC with the left middle cingulate gyrus and left inferior parietal lobule was lower in the RDE group. Compared with the HC group, the FC of the left DC and left inferior parietal lobule was lower in the RDE group (Figure 3C).

One-way ANOVA showed that when the right DC was used as the seed, the right angular was statistically significantly different among the three groups (Table 2, Figure 2E). Compared with the FDE group, the FC of the right DC with the right angular was lower in the RDE group. Compared with the HC group, the FC of the right angular was also lower in the RDE group (Figure 3C).

#### 3.2.3. DCP, DRP, VRP

One-way ANOVA showed that when the left DCP was used as the seed, the right cerebellum anterior lobe was statistically significantly different among the three groups (Table 2, Figure 2F). Compared with the FDE group, the FC of the left DCP with the right cerebellum anterior lobe was lower in the RDE group. Compared with the HC group, the FC of the left DCP with the right cerebellum anterior lobe was also lower in the RDE group (Figure 3D). 

One-way ANOVA showed that when the left DRP was used as the seed, the right caudate and left cerebellum posterior lobe were statistically significantly different among the three groups (Table 2, Figure 2G). Compared with the FDE group, the FC of the left DRP with the right caudate was lower in the RDE group. Compared with the HC group, the FC of the left DRP with the right caudate and left cerebellum posterior lobe was lower in the RDE group, and the FC of the left DRP with the left cerebellum posterior lobe was lower in the FDE group (Figure 3D).

One-way ANOVA showed that when the left VRP was used as the seed, the left cerebellum posterior lobe was statistically significantly different among the three groups (Table 2, Figure 2H). Compared with the FDE group, the FC of the left VRP with the left cerebellum posterior lobe was higher in the RDE group. Compared with the HC group, the FC of the left VRP with the left cerebellum posterior lobe was lower the FDE group (Figure 3D).

### 3.3. Relationship between FC and Clinical Symptoms

**Figure 4:**
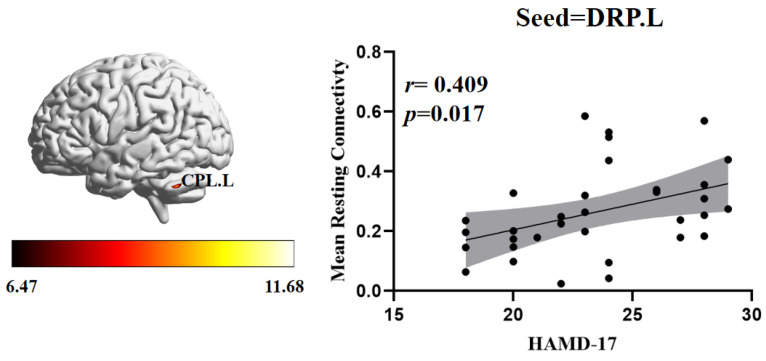
Positive correlation between the FC of abnormal brain regions and the HAMD-17 scores: FC values in the RDE group. CPL.L, left cerebellum posterior lobe; DRP.L, left dorsal rostral putamen.

To investigate the correlation between abnormal brain regions of FC and the severity of clinical depressive symptoms, we further performed a Pearson correlation analysis. In the RDE group, the HAMD-17 scores were positively correlated with the FC between the left DRP and the left cerebellum posterior lobe (*r* = 0.409, *p* = 0.017) (Figure 4).

## 4. Discussion

To our knowledge, this is the first study to analyze differences in striatal whole-brain FC between patients with RDE and FDE. We found FC differences between the RDE and FDE groups in some subregions of the striatum. In addition, the RDE group had more significant alterations in the striatal subregion compared to the HC group. In addition, the HAMD-17 scores were positively correlated with the FC between the left DRP and the left cerebellum posterior lobe in the RDE group

The primary finding of this study was that patients with RDE and FDE had different FC alterations in striatal subregions (VSs.L, DC.L) and DMN. The DMN contains inferior parietal lobule and angular gyrus, which are mainly involved in word comprehension, digit processing and situational memory functions [43,44,45,46]. Previous studies have found abnormal alterations in FC between right DCP and right inferior parietal lobule in insomnia patients compared to the HC group, suggesting that this may be a potential predictor of severity in insomnia patients [42]. 

Other studies have also shown FC disorder between striatum and DMN in patients with MDD [43,44]. A study found hyperconnectivity between VS and ventral DMN but also hypoconnectivity between VS and anterior DMN in MDD patients compared to the HC group. That study also reported that the mechanism of efficacy of electroconvulsive therapy to improve clinical symptoms in MDD patients may be related to the ability to reverse this abnormality [47]. Moreover, another study found abnormalities in FC between the reward network and the DMN in adolescents with depression [48]. A meta-analysis identified lower FC between the reward network and the DMN as an important pathogenesis of RDE, a finding that helps distinguish the RDE group from the HC group [49]. Therefore, the results of this study suggest that abnormal FC alterations between striatal subregions (VSs, DC) and DMN may be an important neuropathological mechanism difference between RDE and FDE, and we speculate that this may be related to the different past medical history of both.

The FC of striatal subregions (VSi.R) and left middle occipital gyrus was higher in the RDE group compared to the FDE group. The middle occipital gyrus, which is mainly associated with the processing of linear space in humans, belongs to the visual processing cortex and plays an important role in the pathogenesis of MDD [50,51,52]. Previous studies have found differences in visual processing cortex function between RDE and FDE, and the regional homogeneity (ReHo) value of left inferior occipital gyrus in the FDE group has been negatively correlated with the HAMD-17 score, suggesting that the left inferior occipital gyrus may be an important neuropathic brain region for FDE [17]. A study found that transcutaneous auricular vagus nerve stimulation increased FC of the right nucleus accumbens and occipital gyrus in patients with MDD [53]. Another study found that acupuncture modulated FC between the striatal subregion and occipital lobe in patients with MDD [54]. These studies suggest that the modulation of the striatal and occipital circuits in patients with MDD may improve MDD. Therefore, the results of this study suggest that FC disorders in the striatum and visual processing cortex are an important mechanism in the pathogenesis of MDD and are important neuropathological circuits that distinguish RDE from FDE.

The cerebellum is involved not only in motor processes but also in cognitive and emotional regulation [55]. Studies have shown that the anterior cerebellar hemisphere is thought to be associated with sensorimotor functions, whereas the posterior cerebellar hemisphere is thought to be associated with emotion, arousal, and cognitive processing of higher order functions in humans [56,57]. Previous studies have shown lower FC in the right VRP and cerebellum posterior lobe in rMDD groups compared to the HC groups, suggesting that lower FC of the striatal-cerebellar is a neural substrate for MDD susceptibility [38]. Another study showed that adolescents with MDD exhibit abnormal functional regulation in frontal-striato-cerebellar regions, suggesting that FC abnormalities of the striatum and cerebellum are important in the pathogenesis of MDD [58]. It was also found that FC between the cerebellum and striatum was higher in the MDD group compared to the HC group, and that ketamine treatment was able to reverse this effect [59]. The results of this study revealed differences between the RDE and FDE groups in the FC of the striatal subregions (VSi.R, DCP.L, DRP.L, VRP.L) and cerebellum, although the differential results were disordered, which may have been related to the longer duration of the disease and the more complex pathological mechanisms in the RDE group. Furthermore, a correlation analysis showed that the FC values of them left DRP and left cerebellum posterior lobe in the RDE group were positively correlated with HAMD-17 scores, suggesting that this abnormal FC may be an important neuropathological mechanism of RDE.

The FC of the striatal subregion (VSs.L, DC.L) with right thalamus, and left middle cingulate gyrus was lower in the RDE group compared to the FDE group. The limbic system is a complex neural network composed of the cingulate gyrus, hippocampus, amygdala, and thalamus that is involved in the generation and expression of emotions in the body, as well as in the formation, storage, and extraction of memories [60,61]. Previous studies have found that intrinsic connections in the prefrontal cortex and striatal-limbic system can distinguish MDD and generalized anxiety disorder, respectively [62]. Another study found that the FC of the right DRP and anterior cingulate gyrus was higher in the rMDD group compared to the acute MDD group, while the FC of the left DCP and thalamus was not significantly different, suggesting that FC in the striatum and limbic system plays an important role in MDD pathogenesis and helps to further differentiate MDD subtypes [38]. The results of this study suggest that FC damage in the striatum and limbic system was more severe in the RDE group compared to the FDE group, which may also be an important therapeutic target for the treatment of RDE.

The results of this study also found that the FC of the right VSs and the left orbital area of the middle frontal gyrus was higher in the RDE group compared to the FDE group, while the FC of the left DRP and the right caudate was lower. The orbitofrontal cortex and caudate are important components of the reward network which are associated with reward motivation and depression [63,64,65]. A previous study found that the FC of the striatal subregion and the putamen in rMDD group was higher compared to that in FDE group, suggesting that FC within the reward network exhibits different FC patterns in different stages of MDD [38]. A review suggests that the orbitofrontal cortico-striato-thalamic circuit plays an important mediating role in the treatment of psychiatric disorders [65]. The results of this study showed that the striatum had lower FC with the reward network in the RDE group compared with the FDE group, which may be an important neuropathological mechanism for the pathogenesis of the RDE group.

In addition, compared with the HC group, we found that the abnormal FC alterations in the striatal subregion were more extensive and deeper in the RDE group than FDE group. Compared with the HC group, a study found more extensive FC changes in striatal subregions in the rMDD group than acute MDD group, suggesting that this may be due to caused by MDD-relieving scars [38]. Another study showed that ReHo and amplitude of low-frequency fluctuations abnormalities were more widely distributed in the RDE group compared with the FDE group, suggesting a more complex neuropathological mechanism in the RDE group [17]. Therefore, we speculate that the results of this study may be related to factors such as recurrent episodes, longer duration of disease and premedication in the RDE group.

## 5. Limitations

Some limitations of this study should be considered. First, we cannot exclude the potential effect of antidepressant factors on brain function in patients with RDE. Second, some of the RDE patients in this study were not first-time relapses, and a study of first-time relapses in RDE patients may be of more clinical research value. Third, this study used GRF correction with a weaker statistical threshold setting of *p* < 0.005. A threshold value of *p* < 0.001 would be more clinically significant for such a study. In the future, the sample size will be expanded, and a more rigorous correction method will be used to improve the scientific value of this study. Finally, HAMD-17 was only used on one clinical scale in this study. In future studies, clinical scales such as anxiety, insomnia, and redundancy will be used to further observe the correlation between abnormal changes in the RDE and FDE striatal circuits and clinical symptoms.

## 6. Conclusions

The results of the present study suggest differences between RDE and FDE patients at the level of striatal circuits, especially related to alterations in the DMN, visual processing cortex, cerebellar sensorimotor network, limbic system and reward network of FC with different striatal areas. This study provides new insights into understanding the specificity of striatal circuits in the RDE group.

## Figures and Tables

**Table 1:** Demographic and clinical characteristics of the study participants.

Variable	RDE (*n* = 33)	FDE (*n* = 27)	HCs (*n* = 35)	*t(F)/χ*2	*p*-Value
Sex (M/F)	8/25	5/22	9/26	0.477	0.788 ^a^
Age (years)	34.63 ± 12.80	33.92 ± 12.36	35.00 ± 12.50	0.056	0.945 ^b^
Years of education	14.39 ± 2.76	14.88 ± 2.37	14.65 ± 3.69	0.198	0.821 ^b^
Duration of illness (months)	24.93 ± 12.27	2.37 ± 0.96	NA	9.513	<0.001 ^c^*
HAMD-17 score	23.48 ± 3.51	22.96 ± 3.36	NA	0.583	0.562 ^c^

RDE, Recurrent depressive episode; FDE, First depressive episode; HCs, Healthy controls; HAMD-17, 17-item Hamilton Rating Scale for Depression; NA, not applicable. ^a^ chi-square test. ^b^ one-way analysis of variance tests. ^c^ two-sample *t*-test. * Significant difference.

**Table 2:** Among three Group Differences in Striatal FC.

Clusters	Brain Regions	MNI Peak			Cluster Size	*F*-Value (Peak)
		X	Y	Z		
**VSi.R**						
1	Left middle occipital gyrus	−50	−76	9	548	10.056
2	Right cerebellum posterior lobe	36	−75	−24	314	14.957
**VSs.L**						
1	Right thalamus	14	−13	9	32	7.980
2	Left inferior parietal lobule	−39	−42	39	37	10.062
**VSs.R**						
1	Left orbital area of the middle frontal gyrus	−3	36	−13	46	9.606
**DC.L**						
1	Left middle cingulate gyrus	−3	−27	39	67	10.631
2	Left inferior parietal lobule	−48	−48	57	45	10.764
**DC.R**						
1	Right angular	42	−57	54	57	10.566
**DCP.L**						
1	Right cerebellum anterior lobe	12	−42	15	69	12.288
**DRP.L**						
1	Right caudate	15	18	6	42	10.868
2	Left cerebellum posterior lobe	−45	−57	−36	45	11.681
**VRP.L**						
1	Left cerebellum posterior lobe	−36	−54	−54	40	11.163

MNI Peak, Coordinates of primary peak locations in the Montreal Neurological Institute space.

## Data Availability

Data can be made available upon reasonable request.

## References

[1] Smith K. (2014). Mental health: A world of depression. Nature.

[2] Zhang F.-F., Peng W., Sweeney J.A., Jia Z.-Y., Gong Q.-Y. (2018). Brain structure alterations in depression: Psychoradiological evidence. CNS Neurosci. Ther..

[3] Friedrich M.J. (2017). Depression Is the Leading Cause of Disability Around the World. JAMA.

[4] Hiller W., Dichtl G., Hecht H., Hundt W., Mombour W., von Zerssen D. (1994). Evaluating the new ICD-10 categories of depressive episode and recurrent depressive disorder. J. Affect. Disord..

[5] Gotlib I.H., Goodman S.H., Humphreys K.L. (2020). Studying the Intergenerational Transmission of Risk for Depression: Current Status and Future Directions. Curr. Dir. Psychol. Sci..

[6] Kessler R.C., Zhao S., Blazer D.G., Swartz M. (1997). Prevalence, correlates, and course of minor depression and major depression in the national comorbidity survey. J. Affect. Disord..

[7] Roca M., Armengol S., García-García M., Rodriguez-Bayón A., Ballesta I., Serrano M.J., Comas A., Gili M. (2011). Clinical differences between first and recurrent episodes in depressive patients. Compr. Psychiatry.

[8] Varghese S., Frey B.N., Schneider M.A., Kapczinski F., Cardoso T.D.A. (2022). Functional and cognitive impairment in the first episode of depression: A systematic review. Acta Psychiatr. Scand..

[9] Zu S., Wang D., Fang J., Xiao L., Zhu X., Wu W., Wang G., Hu Y. (2021). Comparison of Residual Depressive Symptoms, Functioning, and Quality of Life Between Patients with Recurrent Depression and First Episode Depression After Acute Treatment in China. Neuropsychiatr. Dis. Treat..

[10] Gili M., Armengol S., Soriano J.B., Roca M., Garcia-Toro M., Garcia-Campayo J., Vives M. (2011). Medical comorbidity in recurrent versus first-episode depressive patients. Acta Psychiatr. Scand..

[11] Mei L., Wang Y., Liu C., Mou J., Yuan Y., Qiu L., Gong Q. (2022). Study of Sex Differences in Unmedicated Patients with Major Depressive Disorder by Using Resting State Brain Functional Magnetic Resonance Imaging. Front. Neurosci..

[12] Zheng R., Chen Y., Jiang Y., Wen M., Zhou B., Li S., Wei Y., Yang Z., Wang C., Cheng J. (2021). Dynamic Altered Amplitude of Low-Frequency Fluctuations in Patients with Major Depressive Disorder. Front. Psychiatry.

[13] Ma L., Liu M., Xue K., Ye C., Man W., Cheng M., Liu Z., Zhu D., Liu F., Wang J. (2022). Abnormal Regional Spontaneous Brain Activities in White Matter in Patients with Autism Spectrum Disorder. Neuroscience.

[14] Zhao C., Zhu J., Liu X., Pu C., Lai Y., Chen L., Yu X., Hong N. (2018). Structural and functional brain abnormalities in schizophrenia: A cross-sectional study at different stages of the disease. Prog. Neuro-Psychopharmacol. Biol. Psychiatry.

[15] Yan W., Palaniyappan L., Liddle P.F., Rangaprakash D., Wei W., Deshpande G. (2022). Characterization of Hemodynamic Alterations in Schizophrenia and Bipolar Disorder and Their Effect on Resting-State fMRI Functional Connectivity. Schizophr. Bull..

[16] Guo W.-B., Liu F., Xue Z.-M., Xu X.-J., Wu R.-R., Ma C.-Q., Wooderson S.C., Tan C.-L., Sun X.-L., Chen J.-D. (2012). Alterations of the amplitude of low-frequency fluctuations in treatment-resistant and treatment-response depression: A resting-state fMRI study. Prog. Neuro-Psychopharmacol. Biol. Psychiatry.

[17] Sun J., Chen L., He J., Du Z., Ma Y., Wang Z., Guo C., Luo Y., Gao D., Hong Y. (2022). Altered Brain Function in First-Episode and Recurrent Depression: A Resting-State Functional Magnetic Resonance Imaging Study. Front. Neurosci..

[18] Yi L., Wang J., Jia L., Zhao Z., Lu J., Li K., Jia J., He Y., Jiang C., Han Y. (2012). Structural and Functional Changes in Subcortical Vascular Mild Cognitive Impairment: A Combined Voxel-Based Morphometry and Resting-State fMRI Study. PLoS ONE.

[19] Kelly C., Castellanos F. (2014). Strengthening Connections: Functional Connectivity and Brain Plasticity. Neuropsychol. Rev..

[20] Peng X., Wu X., Gong R., Yang R., Wang X., Zhu W., Lin P. (2021). Sub-regional anterior cingulate cortex functional connectivity revealed default network subsystem dysfunction in patients with major depressive disorder. Psychol. Med..

[21] Zhang Y., Shao J., Wang X., Chen Z., Liu H., Pei C., Zhang S., Yao Z., Lu Q. (2021). Functional impairment-based segmentation of anterior cingulate cortex in depression and its relationship with treatment effects. Hum. Brain Mapp..

[22] Luo L., Wu H., Xu J., Chen F., Wu F., Wang C., Wang J. (2021). Abnormal large-scale resting-state functional networks in drug-free major depressive disorder. Brain Imaging Behav..

[23] Yan C.-G., Chen X., Li L., Castellanos F.X., Bai T.-J., Bo Q.-J., Cao J., Chen G.-M., Chen N.-X., Chen W. (2019). Reduced default mode network functional connectivity in patients with recurrent major depressive disorder. Proc. Natl. Acad. Sci. USA.

[24] Roberts H., Jacobs R.H., Bessette K.L., Crowell S.E., Westlund-Schreiner M., Thomas L., Easter R.E., Pocius S.L., Dillahunt A., Frandsen S. (2021). Mechanisms of rumination change in adolescent depression (RuMeChange): Study protocol for a randomised controlled trial of rumination-focused cognitive behavioural therapy to reduce ruminative habit and risk of depressive relapse in high-ruminating adolescents. BMC Psychiatry.

[25] Jacobs R.H., Barba A., Gowins J.R., Klumpp H., Jenkins L.M., Mickey B.J., Ajilore O., Peciña M., Sikora M., Ryan K.A. (2016). Decoupling of the amygdala to other salience network regions in adolescent-onset recurrent major depressive disorder. Psychol. Med..

[26] Friedman E. (1994). CSPT circuity in affective disorders. Biol. Psychiatry.

[27] Bora E., Harrison B., Davey C.G., Yücel M., Pantelis C. (2012). Meta-analysis of volumetric abnormalities in cortico-striatal-pallidal-thalamic circuits in major depressive disorder. Psychol. Med..

[28] Epstein J., Pan H., Kocsis J.H., Yang Y., Butler T., Chusid J., Hochberg H., Murrough J., Strohmayer E., Stern E. (2006). Lack of Ventral Striatal Response to Positive Stimuli in Depressed Versus Normal Subjects. Am. J. Psychiatry.

[29] Gabbay V., Mao X., Klein R.G., Ely B.A., Babb J., Panzer A.M., Alonso C.M., Shungu D.C. (2012). Anterior Cingulate Cortexγ-Aminobutyric Acid in Depressed Adolescents: Relationship to anhedonia. Arch. Gen. Psychiatry.

[30] Zhu X., Helpman L., Papini S., Schneier F., Markowitz J.C., Van Meter P.E., Lindquist M.A., Wager T.D., Neria Y. (2017). Altered resting state functional connectivity of fear and reward circuitry in comorbid PTSD and major depression. Depress. Anxiety.

[31] Gong L., Yin Y., He C., Ye Q., Bai F., Yuan Y., Zhang H., Lv L., Zhang H., Xie C. (2017). Disrupted reward circuits is associated with cognitive deficits and depression severity in major depressive disorder. J. Psychiatr. Res..

[32] Gabbay V., Ely B.A., Li Q., Bangaru S.D., Panzer A.M., Alonso C.M., Castellanos F.X., Milham M.P. (2013). Striatum-Based Circuitry of Adolescent Depression and Anhedonia. J. Am. Acad. Child Adolesc. Psychiatry.

[33] Rutledge R.B., Moutoussis M., Smittenaar P., Zeidman P., Taylor T., Hrynkiewicz L., Lam J., Skandali N., Siegel J.Z., Ousdal O.T. (2017). Association of Neural and Emotional Impacts of Reward Prediction Errors with Major Depression. JAMA Psychiatry.

[34] Hamilton J.P., Sacchet M.D., Hjørnevik T., Chin F.T., Bin Shen B., Kämpe R., Park J.H., Knutson B.D., Williams L.M., Borg N. (2018). Striatal dopamine deficits predict reductions in striatal functional connectivity in major depression: A concurrent 11C-raclopride positron emission tomography and functional magnetic resonance imaging investigation. Transl. Psychiatry.

[35] Pizzagalli D.A., Berretta S., Wooten D., Goer F., Pilobello K.T., Kumar P., Murray L., Beltzer M., Boyer-Boiteau A., Alpert N. (2019). Assessment of Striatal Dopamine Transporter Binding in Individuals with Major Depressive Disorder: In Vivo Positron Emission Tomography and Postmortem Evidence. JAMA Psychiatry.

[36] Peciña M., Sikora M., Avery E.T., Heffernan J., Peciña S., Mickey B.J., Zubieta J.-K. (2017). Striatal dopamine D2/3 receptor-mediated neurotransmission in major depression: Implications for anhedonia, anxiety and treatment response. Eur. Neuropsychopharmacol..

[37] Di Martino A., Scheres A., Margulies D., Kelly C., Uddin L., Shehzad Z., Biswal B., Walters J.R., Castellanos F., Milham M.P. (2008). Functional Connectivity of Human Striatum: A Resting State fMRI Study. Cereb. Cortex.

[38] Wang L., Li F., Mitchell P.B., Wang C.-Y., Si T.-M. (2020). Striatal Resting-State Connectivity Abnormalities Associated with Different Clinical Stages of Major Depressive Disorder. J. Clin. Psychiatry.

[39] Admon R., Holsen L.M., Aizley H., Remington A., Whitfield-Gabrieli S., Goldstein J.M., Pizzagalli D.A. (2015). Striatal Hypersensitivity During Stress in Remitted Individuals with Recurrent Depression. Biol. Psychiatry.

[40] Hammar Å., Neto E., Clemo L., Hjetland G.J., Hugdahl K., Elliott R. (2016). Striatal hypoactivation and cognitive slowing in patients with partially remitted and remitted major depression. PsyCh J..

[41] Chao-Gan Y., Yu-Feng Z. (2010). DPARSF: A MATLAB toolbox for “pipeline” data analysis of resting-state fMRI. Front. Syst. Neurosci..

[42] Wang L., Wang K., Liu J.-H., Wang Y.-P. (2018). Altered Default Mode and Sensorimotor Network Connectivity with Striatal Subregions in Primary Insomnia: A Resting-State Multi-Band fMRI Study. Front. Neurosci..

[43] Raichle M.E., MacLeod A.M., Snyder A.Z., Powers W.J., Gusnard D.A., Shulman G.L. (2001). A default mode of brain function. Proc. Natl. Acad. Sci. USA.

[44] Buckner R.L., Andrews-Hanna E.J.R., Schactera D.L. (2008). The Brain’s Default Network: Anatomy, Function, and Relevance to Disease. Ann. N. Y. Acad. Sci..

[45] Yamamura T., Okamoto Y., Okada G., Takaishi Y., Takamura M., Mantani A., Kurata A., Otagaki Y., Yamashita H., Yamawaki S. (2016). Association of thalamic hyperactivity with treatment-resistant depression and poor response in early treatment for major depression: A resting-state fMRI study using fractional amplitude of low-frequency fluctuations. Transl. Psychiatry.

[46] Seghier M.L. (2013). The Angular Gyrus: Multiple functions and multiple subdivisions. Neuroscientist.

[47] Leaver A.M., Espinoza R., Joshi S.H., Vasavada M., Njau S., Woods R.P., Narr K.L. (2016). Desynchronization and Plasticity of Striato-frontal Connectivity in Major Depressive Disorder. Cereb. Cortex.

[48] Ho T.C., Connolly C.G., Blom E.H., LeWinn K.Z., Strigo I.A., Paulus M.P., Frank G., Max J.E., Wu J., Chan M. (2015). Emotion-Dependent Functional Connectivity of the Default Mode Network in Adolescent Depression. Biol. Psychiatry.

[49] Ding Y.-D., Chen X., Chen Z.-B., Le Li L., Li X.-Y., Castellanos F.X., Bai T.-J., Bo Q.-J., Cao J., Chang Z.-K. (2022). Reduced nucleus accumbens functional connectivity in reward network and default mode network in patients with recurrent major depressive disorder. Transl. Psychiatry.

[50] Renier L.A., Anurova I., De Volder A.G., Carlson S., VanMeter J., Rauschecker J.P. (2010). Preserved Functional Specialization for Spatial Processing in the Middle Occipital Gyrus of the Early Blind. Neuron.

[51] Geng J., Yan R., Shi J., Chen Y., Mo Z., Shao J., Wang X., Yao Z., Lu Q. (2019). Altered regional homogeneity in patients with somatic depression: A resting-state fMRI study. J. Affect. Disord..

[52] Teng C., Zhou J., Ma H., Tan Y., Wu X., Guan C., Qiao H., Li J., Zhong Y., Wang C. (2018). Abnormal resting state activity of left middle occipital gyrus and its functional connectivity in female patients with major depressive disorder. BMC Psychiatry.

[53] Wang Z., Fang J., Liu J., Rong P., Jorgenson K., Park J., Lang C., Hong Y., Zhu B., Kong J. (2018). Frequency-dependent functional connectivity of the nucleus accumbens during continuous transcutaneous vagus nerve stimulation in major depressive disorder. J. Psychiatr. Res..

[54] Wang Z., Wang X., Liu J., Chen J., Liu X., Nie G., Jorgenson K., Sohn K.C., Huang R., Liu M. (2017). Acupuncture treatment modulates the corticostriatal reward circuitry in major depressive disorder. J. Psychiatr. Res..

[55] Van Overwalle F., Manto M., Cattaneo Z., Clausi S., Ferrari C., Gabrieli J.D.E., Guell X., Heleven E., Lupo M., Ma Q. (2020). Consensus Paper: Cerebellum and Social Cognition. Cerebellum.

[56] Olivito G., Lupo M., Iacobacci C., Clausi S., Romano S., Masciullo M., Molinari M., Cercignani M., Bozzali M., Leggio M. (2018). Structural cerebellar correlates of cognitive functions in spinocerebellar ataxia type 2. J. Neurol..

[57] Depping M.S., Schmitgen M.M., Kubera K.M., Wolf R.C. (2018). Cerebellar Contributions to Major Depression. Front. Psychiatry.

[58] Chantiluke K., Halari R., Simic M., Pariante C.M., Papadopoulos A., Giampietro V., Rubia K. (2012). Fronto-Striato-Cerebellar Dysregulation in Adolescents with Depression during Motivated Attention. Biol. Psychiatry.

[59] Sahib A.K., Loureiro J.R., Vasavada M., Anderson C., Kubicki A., Wade B., Joshi S.H., Woods R.P., Congdon E., Espinoza R. (2020). Modulation of the functional connectome in major depressive disorder by ketamine therapy. Psychol. Med..

[60] Rolls E.T. (2019). The cingulate cortex and limbic systems for action, emotion, and memory. Handb. Clin. Neurol..

[61] Heimer L., Van Hoesen G.W. (2006). The limbic lobe and its output channels: Implications for emotional functions and adaptive behavior. Neurosci. Biobehav. Rev..

[62] Xu X., Dai J., Chen Y., Liu C., Xin F., Zhou X., Zhou F., Stamatakis E.A., Yao S., Luo L. (2021). Intrinsic connectivity of the prefrontal cortex and striato-limbic system respectively differentiate major depressive from generalized anxiety disorder. Neuropsychopharmacology.

[63] Hu H. (2016). Reward and Aversion. Annu. Rev. Neurosci..

[64] Lacerda A.L., Keshavan M.S., Hardan A.Y., Yorbik O., Brambilla P., Sassi R.B., Nicoletti M., Mallinger A.G., Frank E., Kupfer D.J. (2004). Anatomic evaluation of the orbitofrontal cortex in major depressive disorder. Biol. Psychiatry.

[65] Fettes P., Schulze L., Downar J. (2017). Cortico-Striatal-Thalamic Loop Circuits of the Orbitofrontal Cortex: Promising Therapeutic Targets in Psychiatric Illness. Front. Syst. Neurosci..

